# Phloroglucinol-Gold and -Zinc Oxide Nanoparticles: Antibiofilm and Antivirulence Activities towards *Pseudomonas aeruginosa* PAO1

**DOI:** 10.3390/md19110601

**Published:** 2021-10-22

**Authors:** Fazlurrahman Khan, Min-Gyun Kang, Du-Min Jo, Pathum Chandika, Won-Kyo Jung, Hyun Wook Kang, Young-Mog Kim

**Affiliations:** 1 Research Center for Marine Integrated Bionics Technology, Pukyong National University, Busan 48513, Korea; fkhan055@pknu.ac.kr (F.K.); wkjung@pknu.ac.kr (W.-K.J.); 2 Department of Food Science and Technology, Pukyong National University, Busan 48513, Korea; hghgh552@naver.com (M.-G.K.); dmin6778@gmail.com (D.-M.J.); 3 Department of Biomedical Engineering and New-Senior Healthcare Innovation Center (BK21 Plus), Pukyong National University, Busan 48513, Korea; pdpatabedi@gmail.com; 4 Department of Biomedical Engineering, Pukyong National University, Busan 48513, Korea; wkang@pukyong.ac.kr; 5 Industry 4.0 Convergence Bionics Engineering, Pukyong National University, Busan 48513, Korea

**Keywords:** antibiofilm, antivirulence, phloroglucinol, PG-AuNPs, PG-ZnONPs, nanoparticles, *Pseudomonas aeruginosa*

## Abstract

With the advancement of nanotechnology, several nanoparticles have been synthesized as antimicrobial agents by utilizing biologically derived materials. In most cases, the materials used for the synthesis of nanoparticles from natural sources are extracts. Natural extracts contain a wide range of bioactive components, making it difficult to pinpoint the exact component responsible for nanoparticle synthesis. Furthermore, the bioactive component present in the extract changes according to numerous environmental factors. As a result, the current work intended to synthesize gold (AuNPs) and zinc oxide (ZnONPs) nanoparticles using pure phloroglucinol (PG). The synthesized PG-AuNPs and PG-ZnONPs were characterized using a UV–Vis absorption spectrophotometer, FTIR, DLS, FE-TEM, zeta potential, EDS, and energy-dispersive X-ray diffraction. The characterized PG-AuNPs and PG-ZnONPs have been employed to combat the pathogenesis of *Pseudomonas aeruginosa*. *P*. *aeruginosa* is recognized as one of the most prevalent pathogens responsible for the common cause of nosocomial infection in humans. Antimicrobial resistance in *P*. *aeruginosa* has been linked to the development of recalcitrant phenotypic characteristics, such as biofilm, which has been identified as one of the major obstacles to antimicrobial therapy. Furthermore, *P*. *aeruginosa* generates various virulence factors that are a major cause of chronic infection. These PG-AuNPs and PG-ZnONPs significantly inhibit early stage biofilm and eradicate mature biofilm. Furthermore, these NPs reduce *P*. *aeruginosa* virulence factors such as pyoverdine, pyocyanin, protease, rhamnolipid, and hemolytic capabilities. In addition, these NPs significantly reduce *P*. *aeruginosa* swarming, swimming, and twitching motility. PG-AuNPs and PG-ZnONPs can be used as control agents for infections caused by the biofilm-forming human pathogenic bacterium *P*. *aeruginosa*.

## 1. Introduction

*Pseudomonas aeruginosa* is commonly isolated from individuals with severe medical illnesses such as cystic fibrosis, bacteremia, severe burns, immunological dysfunction, and respiratory infections [1,2]. Furthermore, it has been known to be pathogenic to plants, animals, and invertebrates [3]. It is highly adaptable to severe environmental conditions and possesses intrinsic/acquired antibiotic resistance mechanisms [4,5]. Survivability and other resistance properties were also observed as a result of biofilm formation, which has been classified as an adaptive resistance mechanism [6]. *P*. *aeruginosa* biofilm formation has been observed on the surfaces of biotic, abiotic, and biomedical equipment [7]. It has also been reported that, during chronic infection, it can form biofilms on the surface of host tissues/epithelia [2,8]. Biofilms provide protection against host immune responses in addition to functioning as a barrier to antimicrobial drugs [8]. Furthermore, chronic infections caused by *P*. *aeruginosa* have been identified due to the production of several virulence factors such as protease, elastase, pyocyanin, siderophores, phospholipase C, exotoxin A, rhamnolipids, exoenzymes S, and the *Pseudomonas* quinolone signal [4,9]. Furthermore, different types of motility, such as swarming, swimming, and twitching, were involved in infection, colonization, and adhesion to biotic and abiotic surfaces [10]. In addition, the biofilm environment serves as a reservoir for antibiotic-tolerant persister cells, which are responsible for chronic and recurring infection [11]. As a result of its opportunistic pathogenicity and production of diverse virulence factors, biofilm formation, and generation of persister cells, *P*. *aeruginosa* has become one of the model organisms for the study of pathogenesis, antimicrobial resistance, and the discovery of antimicrobial treatment strategies [12]. Nanotechnology has emerged as a promising technology for combating microbial infection through the use of metal- and metal-oxide-based nanoparticles. Currently, a variety of metallic (e.g., gold and silver) and metal oxide (ZnO, Co, and CeO_2_) nanoparticles for treating bacterial infections have been synthesized by utilizing naturally derived extracts (from algae, bacteria, fungi, and plants) [13,14,15,16,17,18,19]. Green synthesis of nanoparticles by utilizing biologically derived materials is a promising approach compared to physical and chemical methods because it is easy, cost-effective, eco-friendly, and reduces the risk of toxic residues being released into the environment [20,21]. Furthermore, nanoparticles synthesized using the green synthesis approach are stable and biocompatible [20,22]. Some of the unique properties of metal nanoparticles, such as a large surface area, surface charge, and small size, lead to a higher strength of cellular interactions [23]. Additionally, metallic nanoparticles are described as an effective drug delivery vehicle with minimal toxicity, specific targeting, and controlled release [23,24]. The mechanism of antimicrobial activity of metal and metal oxide nanoparticles has been well understood, including the generation of cellular reactive oxygen species, cell membrane damage, cell membrane penetration, disruption of signaling pathways, inhibition of protein synthesis, and DNA damage [25,26].

Since naturally derived extracts contain a variety of bioactive components, determining the specific component responsible for the nanoparticle synthesis that utilizes the extract would be challenging. Furthermore, the bioactive component present in the extract changes according to numerous environmental factors. Gold nanoparticles (AuNPs) and zinc oxide nanoparticles (ZnONPs) have been shown to be highly stable, biocompatible, non-toxic to humans, and function as an excellent drug delivery system [27,28]. Moreover, the United States Food and Drug Administration has approved ZnONPs as a safe material [29]. As a result, the current work intended to synthesize gold (AuNPs) and zinc oxide (ZnONPs) nanoparticles using phloroglucinol (PG). PG and its derivatives have been identified as a natural compound from plants, algae, and microbes, and have been shown to have antioxidant, antibacterial, anticancer, anthelminthic, and antidiabetic properties [30,31,32,33,34,35]. The PG-AuNPs and PG-ZnONPs that were synthesized were evaluated as potential antibiofilm and antivirulence agents against *P*. *aeruginosa*.

## 2. Results

### 2.1. Synthesis and Characterization of PG-AuNPs and PG-ZnONPs

**Figure 1:**
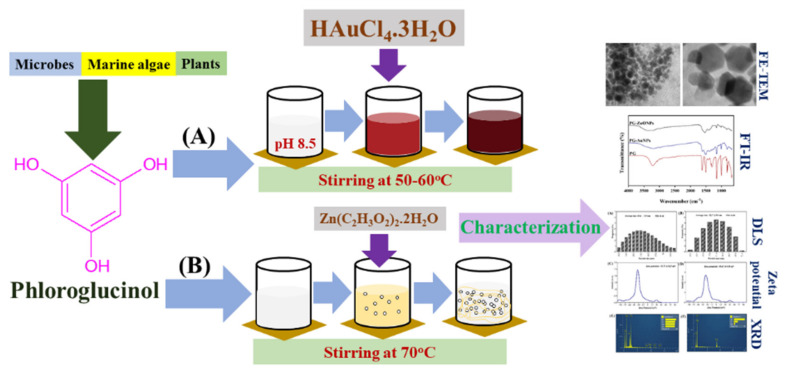
(**A**) Schematic representation for the synthesis of PG-AuNPs and (**B**) PG-ZnONPs. Synthesis of PG-AuNPs was carried out by mixing a PG solution and aqueous gold (III) chloride trihydrate (HAuCl_4_.3H_2_O) with a pH of 8.5–9.0 under stirring conditions at 50–60 °C. Similarly, the synthesis of ZnONPs was carried out by mixing a PG solution with a solution of Zn(CH_3_-COO)_2_.2H_2_O (20 mM) under continuous stirring conditions at 70 °C.

Two types of nanoparticles have been synthesized from PG: one is metallic, such as gold nanoparticles (PG-AuNPs), and the other is a metal oxide, such as zinc oxide nanoparticles (PG-ZnONPs). Figure 1 depicts a schematic representation of the steps involved in the synthesis of PG-AuNPs (A) and PG-ZnONPs (B).

**Figure 2:**
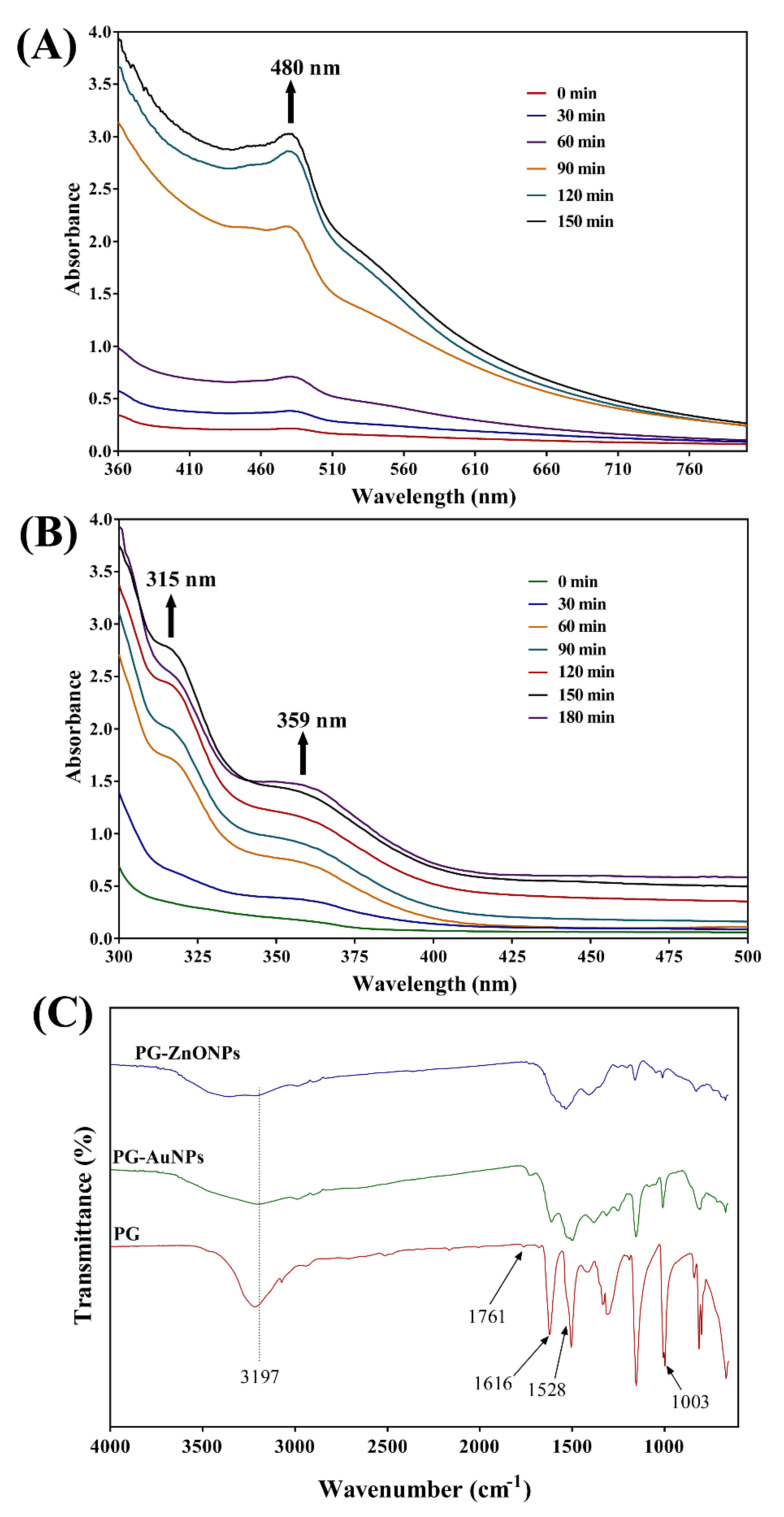
(**A**) UV–Vis absorption spectra of PG-AuNPs, (**B**) UV–Vis absorption spectra of PG-ZnONPs, and (**C**) FTIR spectra of PG, PG-AuNPs, and PG-ZnONPs.

The formation of PG-AuNPs was confirmed by the apparent change in color from yellow to wine-red. Furthermore, the formation of PG-AuNPs was verified by analyzing UV–Vis absorption spectra at different time intervals throughout the chemical reaction. The maximum absorption peak of PG-AuNPs was observed at 480 nm (Figure 2A). Similarly, visual observation of the white precipitate formation confirmed the formation of PG-ZnONPs. Additionally, the absorption spectra analysis revealed that PG-ZnONPs have two distinct absorption peaks, one at 315 nm and the other at 359 nm (Figure 2B). Furthermore, FTIR analysis was used to validate the synthesis of PG-AuNPs and PG-ZnONPs (Figure 2C).

**Figure 3:**
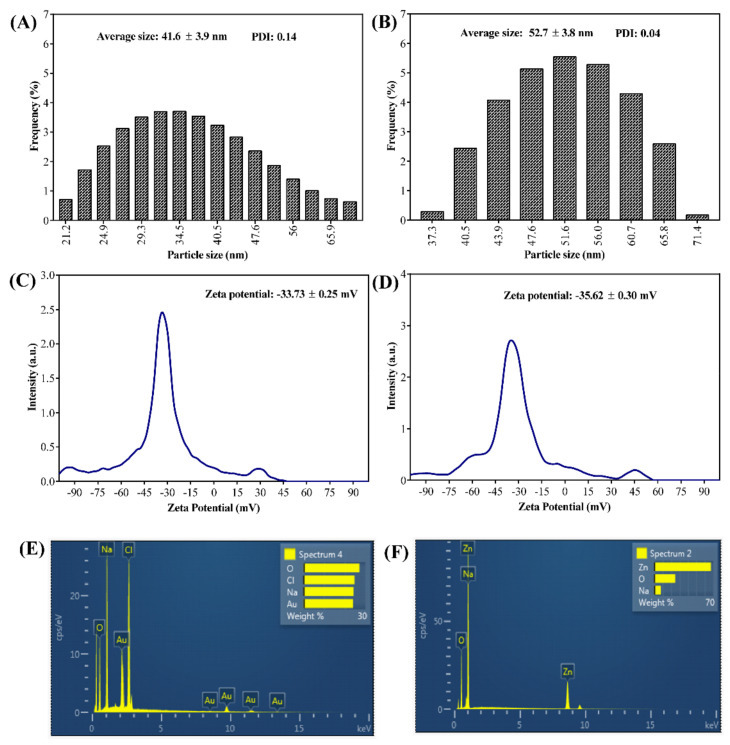
(**A**) Dynamic light scattering (DLS) particle size distribution of PG-AuNPs, (**B**) dynamic light scattering (DLS) particle size distribution of PG-ZnONPs, (**C**) zeta potential of PG-AuNPs, (**D**) zeta potential of PG-ZnONPs, (**E**) energy-dispersive X-ray diffraction (EDS) spectrum of PG-AuNPs, and (**F**) energy-dispersive X-ray diffraction (EDS) spectrum of PG-ZnONPs.

**Figure 4:**
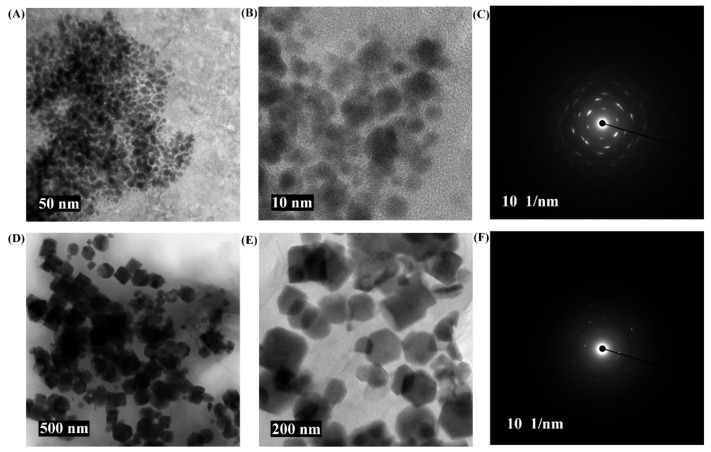
FE-TEM micrograph of PG-AuNPs and PG-ZnONPs. (**A**) FE-TEM micrograph of PG-AuNPs at a resolution of 50 nm, (**B**) FE-TEM micrograph of PG-AuNPs at a resolution of 10 nm, (**C**) SAED of PG-AuNPs, (**D**) FE-TEM micrograph of PG-ZnONPs at a resolution of 500 nm, (**E**) FE-TEM micrograph of PG-ZnONPs at a resolution of 200 nm, and (**F**) SAED of PG-ZnONPs.

The FTIR analysis revealed that the pure PG compound shows some characteristic vibration bands, as seen at 3197, 1528, and 1003 cm^−1^, which correspond to v(OH), δ(C=C), and δ(OH). The FTIR spectra of PG-AuNPs and PG-ZnONPs differed from those of the pure PG compound. Interestingly, some of the characteristic vibration bands of PG have been observed in the FTIR spectra of PG-AuNPs and PG-ZnONPs, while others have vanished (Figure 2C). The appearance and disappearance of some characteristic vibration bands in PG-AuNPs and PG-ZnONPs indicated that PG interacted with AuNPs and ZnONPs. Dynamic light scattering (DLS) was used to determine the size of PG-AuNPs and PG-ZnONPs, and the particle size distribution of PG-AuNPs and PG-ZnONPs is shown in Figure 3A,B. The average particle size and polydispersity index (PDI) of PG-AuNPs were 41.6 ± 3.9 nm and 0.14, respectively (Figure 3A). Similarly, the average particle size and PDI value of PG-ZnONPs were found to be 52.7 ± 3.8 nm and 0.04, respectively (Figure 3B). FE-TEM revealed that the shape of PG-AuNPs was spherical (Figure 4A,B). The predominant shape of PG-ZnONPs was hexagonal; however, other shapes have also been reported (Figure 4D,E). Determination of surface charge on the PG-AuNPs and PG-ZnONPs was carried out by measuring the zeta potentials. The zeta potential of PG-AuNPs was -33.73 ± 0.25 mV (Figure 3C), whereas the zeta potential of PG-ZnONPs was -35.62 ± 0.30 mV (Figure 3D).

**Figure 5:**
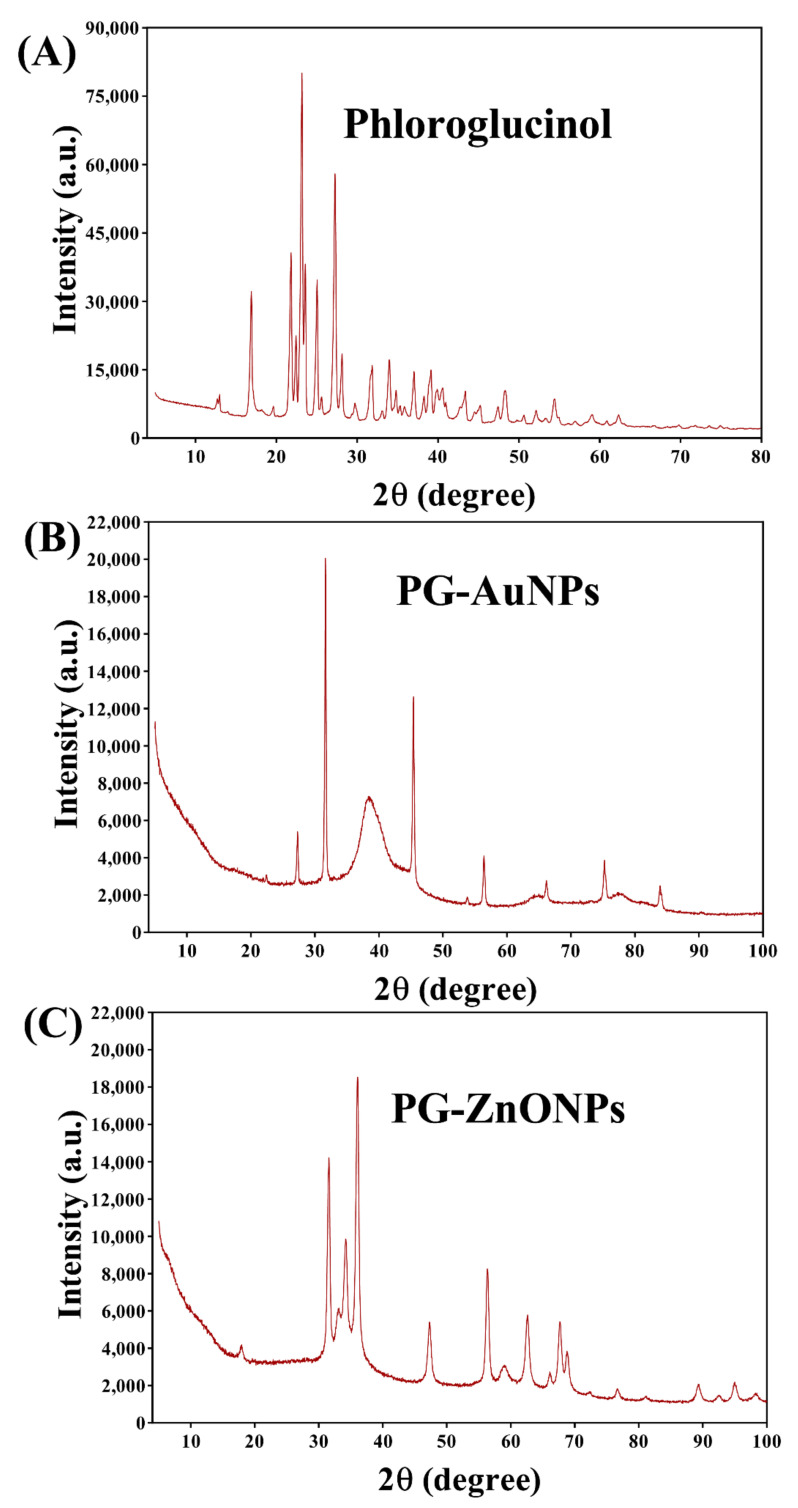
XRD spectra of PG (**A**), XRD spectra of PG-AuNPs (**B**), and XRD- pectra of PG-ZnONPs (**C**).

EDS analysis revealed the presence of Au and Zn atoms in the elemental composition of PG-AuNPs and PG-ZnONPs (Figure 3E,F). XRD was used to examine the crystalline nature of PG-AuNPs and PG-ZnONPs. From 20° to 30°, some of the most prominent and distinctive PG diffraction peaks were identified (Figure 5A). These characteristic peaks of PG were identified at 21.7°, 23.1°, 25°, and 27.2°. The characteristic diffraction peaks of AuNPs, as previously described in several publications, were confirmed to be present in PG-AuNPs (Figure 5B). The diffraction peaks of PG-AuNPs at 2θ were found to be 38.3°, 45.3°, 56.4°, 66.1°, 75.2°, and 83.8°, which were identical to those previously reported for AuNPs. The diffraction peak of the PG-ZnONP investigation revealed the presence of characteristic peaks with 2θ values at 31.6°, 34°, 36°, 47.3°, 56.4°, 62.6°, 67.6°, 68.8°, and 76.6° (Figure 5C). Similar diffraction peaks have previously been reported for ZnONPs synthesized using plant extracts [13]. Furthermore, the presence of the corresponding Debye–Scherrer rings in SAED verified the crystalline nature of these NPs (Figure 4C,F). The characterization of PG-AuNPs and PG-ZnONPs, using multiple instruments, confirmed the successful synthesis of NPs using a green chemistry approach.

### 2.2. Effect of PG, PG-AuNPs, PG-ZnONPs, and C-ZnONPs on the Growth Properties of P. aeruginosa

**Figure 6:**
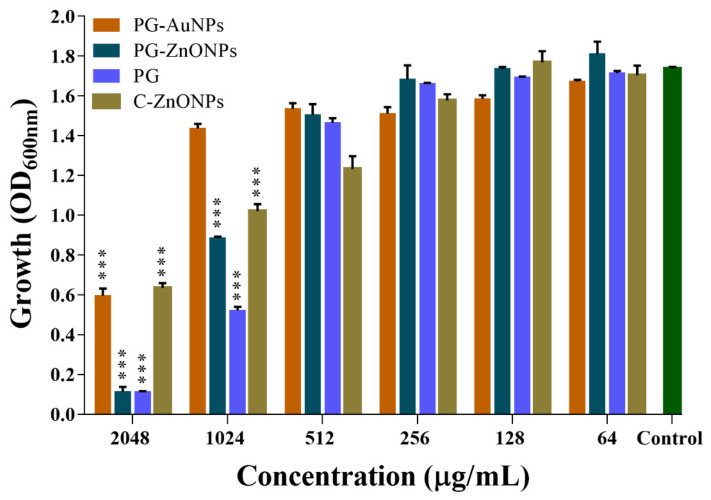
Determination of MIC values of PG-AuNPs, PG-ZnONPs, PG, and C-ZnONPs towards *P*. *aeruginosa*. The MIC value was determined by the microbroth dilution method by incubating the cell culture with different concentrations of NPs. The MIC value was decided based on the OD_600_ values. *** denotes significance at *p* < 0.0001.

The effect of different concentrations (32 to 2048 μg/mL) of PG, PG-AuNPs, PG-ZnONPs, and commercial-ZnONPs (C-ZnONPs) on the cell growth of *P*. *aeruginosa* was studied under shaking conditions, and the OD_600_ was measured after 24 h of incubation. The cell growth of NPs and PG-treated cells differed from that of the control (Figure 6). *P*. *aeruginosa* cell growth was inhibited to 65.66% at the highest concentration of PG-AuNPs (2048 μg/mL) and only 17.25% at a concentration of 1024 μg/mL. However, cell growth was unaffected below a concentration of 1024 μg/mL of PG-AuNPs. At the highest concentration (2048 μg/mL), PG-ZnONPs inhibit cell growth by 93.46%, whereas at 1024 μg/mL they inhibit it by 48.9%. Similar to the PG-AuNPs, PG-ZnONPs had no effect on cell growth at concentrations lower than 1024 μg/mL. C-ZnONPs inhibit cell growth by up to 63.4% at a concentration of 2048 μg/mL. On the other hand, C-ZnONPs show 41.2% inhibition at 1024 μg/mL, which is close to the inhibition observed with PG-ZnONPs at the same concentration (Figure 6). The pure PG compound inhibits cell growth the most (93.37%) at a 2048 μg/mL concentration and only 69.93% at a concentration of 1024 μg/mL. Based on the aforesaid findings, the MIC of PG-ZnONPs and PG was determined to be 2048 μg/mL. On the other hand, the MIC value of PG-AuNPs and C-ZnONPs was determined to be > 2048 μg/mL because, at this concentration, they inhibit cell growth by up to 65.66% and 63.44%, respectively. Since these NPs did not inhibit growth at concentrations lower than 1024 μg/mL, the concentration range of 32 to 512 μg/mL was considered as sub-MIC. This concentration range of NPs was then chosen for evaluating their biofilm and virulence suppression properties in *P*. *aeruginosa*.

### 2.3. PG-AuNPs and PG-ZnONPs Inhibited Biofilm Formation and Eradicated Mature Biofilms

The effect of PG-AuNPs and PG-ZnONPs on the formation of biofilm, which is one of the most important phenotypic characteristics exhibited by *P*. *aeruginosa* in the course of chronic infection, was investigated [26]. Concentrations of PG-AuNPs and PG-ZnONPs were selected in the range of 32 to 512 μg/mL.

**Figure 7:**
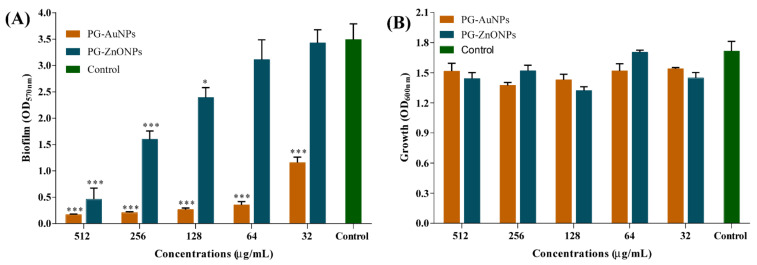
Inhibition of *P*. *aeruginosa* biofilm by PG-AuNPs and PG-ZnONPs. (**A**) Antibiofilm effect of PG-AuNPs and PG-ZnONPs and (**B**) growth properties in the presence of PG-AuNPs and PG-ZnONPs. The biofilm assays were carried out via staining with crystal violet and quantified by measuring the OD at 570 nm. The cell culture (OD 0.05) was treated with different concentrations of NPs and incubated at 37 °C for 24 h. Statistical analysis was carried out using one-way ANOVA, followed by Dunnett’s multiple comparisons test. *** and * denote significance at *p* < 0.0001 and *p* < 0.05, respectively, in one-way ANOVA.

**Figure 8:**
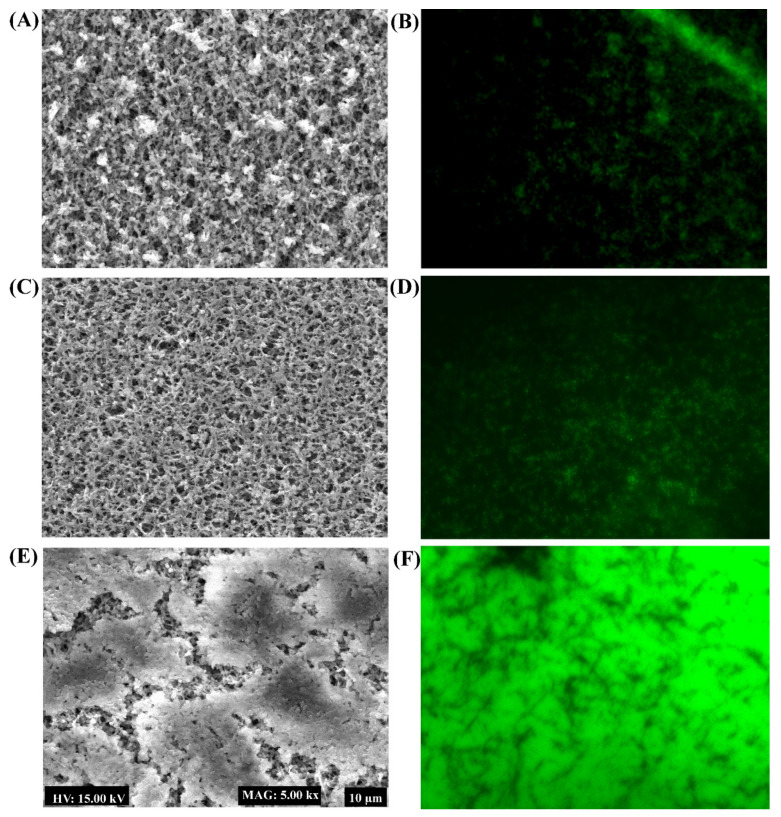
Biofilm architecture analysis of *P*. *aeruginosa* treated with PG-AuNPs and PG-ZnONPs. (**A**) SEM image of the biofilm cells treated with PG-AuNPs (512 μg/mL), (**B**) fluorescent image of the biofilm cells treated with PG-AuNPs (512 μg/mL), (**C**) SEM image of the biofilm cells treated with PG-ZnONPs (512 μg/mL), (**D**) fluorescent image of the biofilm cells treated with PG-ZnONPs (512 μg/mL), (**E**) SEM image of the untreated biofilm cells (control), and (**F**) fluorescent image of the untreated biofilm cells (control).

The biofilm inhibitory effect of PG-AuNPS and PG-ZnONPs was found to act in a concentration-dependent manner (Figure 7A). The highest degree of biofilm inhibition, 95% by PG-AuNPs and 86.8% by PG-ZnONPs, was determined to be at a concentration of 512 μg/mL (Figure 7A). Figure 7B shows the cell growth of *P*. *aeruginosa* during the biofilm assays in the presence of the abovementioned concentration range of PG-AuNPs and PG-ZnONPs. These concentrations did not affect the cell growth, suggesting that the biofilm inhibitory action of these NPs was growth-independent. To verify the biofilm inhibitory effect of PG-AuNPs and PG-ZnONPs, scanning electron microscopy was carried out to determine the biofilm architecture in the presence of these NPs (Figure 8). A compact, dense biofilm architecture was observed in untreated cells (Figure 8E), whereas treated cells with PG-AuNPs (Figure 8A) and PG-ZnONPs (Figure 8C) did not show adhered cells on the surface of the nylon membrane. In addition to the SEM examination, fluorescence microscopy was used to confirm the biofilm inhibitory effect of these NPs (Figure 8B,D,F). The fluorescence intensity was considerably decreased in the PG-AuNP- (Figure 8B) and PG-ZnONP- (Figure 8D) treated cells compared to the control (Figure 8F).

**Figure 9:**
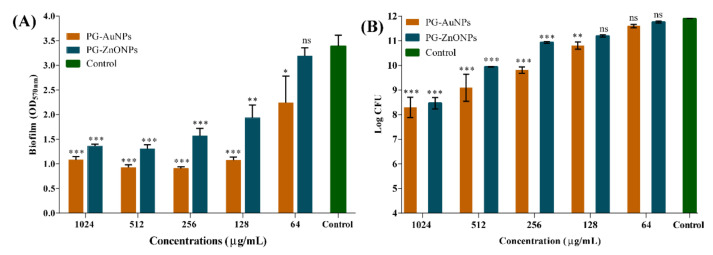
Eradication of *P*. *aeruginosa* mature biofilm by PG-AuNPs and PG-ZnONPs. (**A**) OD_570_ measurements of biofilm cells after staining with crystal violet and (**B**) CFU values of biofilm cells. The mature biofilm was allowed to form by growing the cell culture (OD 0.05). The mature biofilm of the bacterial cell was treated with varying concentrations of PG-AuNPs and PG-ZnONPs, and then incubated at 37 °C. Crystal violet staining and the measuring of CFU values were used to quantify the cells remaining following the dispersion impact of NPs. ***, **, and * denote significance at *p* < 0.0001, *p* < 0.01, and *p* < 0.05, whereas ns denotes non-significance.

To determine the effectiveness of these NPs in eradicating *P*. *aeruginosa* mature biofilms, mature biofilms that had been established for 24 h were treated with various dosages of PG-AuNPs and PG-ZnONPs. The eradication effectiveness was evaluated using both crystal violet staining and colony counting methods. The elimination of mature biofilm by PG-AuNPs and PG-ZnONPs was concentration-dependent (Figure 9A). Biofilm eradication was reported to be 68.1% and 59.8% at the maximum concentration (1024 μg/mL) of PG-AuNPs and PG-ZnONPs, respectively. The eradication effectiveness of PG-AuNPs and PG-ZnONPs, as measured by CFU values, was also concentration-dependent (Figure 9B). At a concentration of 1024 μg/mL, PG-AuNPs and PG-ZnONPs reduce *P*. *aeruginoa* cells from the mature biofilm by 3.6 and 3.4 log CFU, respectively. Thus, the aforementioned findings demonstrate that PG-AuNPs and PG-ZnONPs have biofilm inhibitory properties at the early stage of biofilm formation and may eradicate established, mature *P*. *aeruginosa* biofilms.

### 2.4. Inhibitory Effect of PG-AuNPs and PG-ZnONPs on Virulence Properties

**Figure 10:**
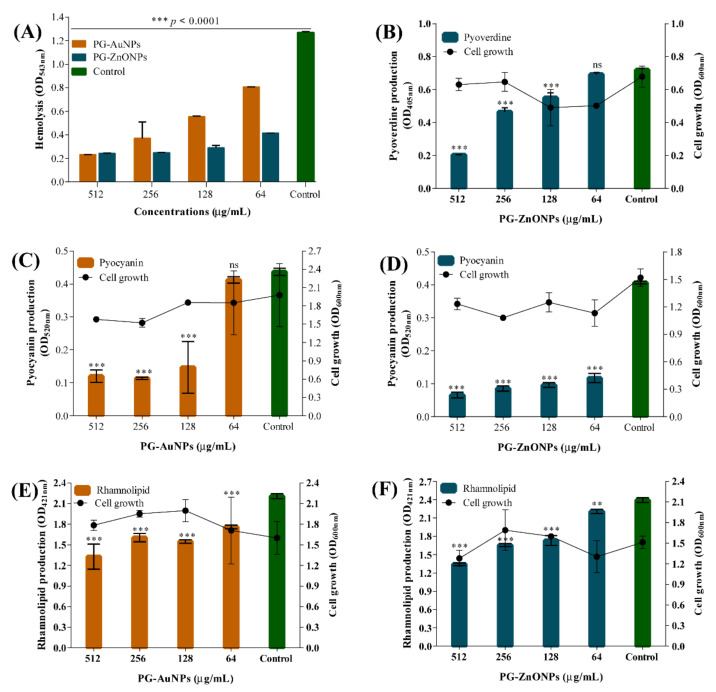
Antivirulence activity of PG-AuNPs and PG-ZnONPs towards *P*. *aeruginosa*. (**A**) Effect of PG-AuNPs and PG-ZnONPs on hemolytic activity, (**B**) effect of PG-ZnONPs on pyoverdine production (**note**: we were unable to determine pyoverdine production in the presence of PG-AuNPs due to its dark-brown color, which obscures the yellow–green color of pyoverdine produced in the colorless MSM), (**C**) effect of PG-AuNPs on pyocyanin production, (**D**) effect of PG-ZnONPs on pyocyanin production, (**E**) effect of PG-AuNPs on rhamnolipid production, and (**F**) effect of PG-ZnONPs on rhamnolipid production. *** and ** denote significance at *p* < 0.0001 and *p* < 0.01, whereas ns denotes non-significance.

The hemolytic property of *P*. *aeruginosa* was significantly affected in the presence of all tested concentrations of PG-AuNPs and PG-ZnONPs (Figure 10A). The hemolytic inhibition by PG-AuNPs was concentration-dependent. The inhibition of hemolytic activity at the sub-MIC concentration (512 μg/mL) of PG-AuNPs and PG-ZnONPs was identified to be 81.7% and 80.7%, respectively. The inhibitory effect of PG-ZnONPs on the pyoverdine siderophore of *P*. *aeruginosa* was concentration-dependent (Figure 10B), with the highest inhibition of 71.7% determined at 512 μg/mL. Unfortunately, we were unable to determine pyoverdine production in the presence of PG-AuNPs due to its dark-brown color, which obscures the yellow–green color of pyoverdine produced in the colorless minimal salt medium (MSM). Both PG-AuNPs and PG-ZnONPs significantly inhibited *P*. *aeruginosa* pyocyanin production (Figure 10C,D). The inhibition of pyocyanin production by PG-AuNPs and PG-ZnONPs at sub-MIC concentrations (512 μg/mL) was 72.5% and 83.8%, respectively. Similarly, PG-AuNPs and PG-ZnONPs significantly inhibited *P*. *aeruginosa* rhamnolipid production (Figure 10E,F). At sub-MIC concentrations (512 μg/mL), PG-AuNPs and PG-ZnONPs inhibited rhamnolipid production by 39.8% and 43.9%, respectively.

**Figure 11:**
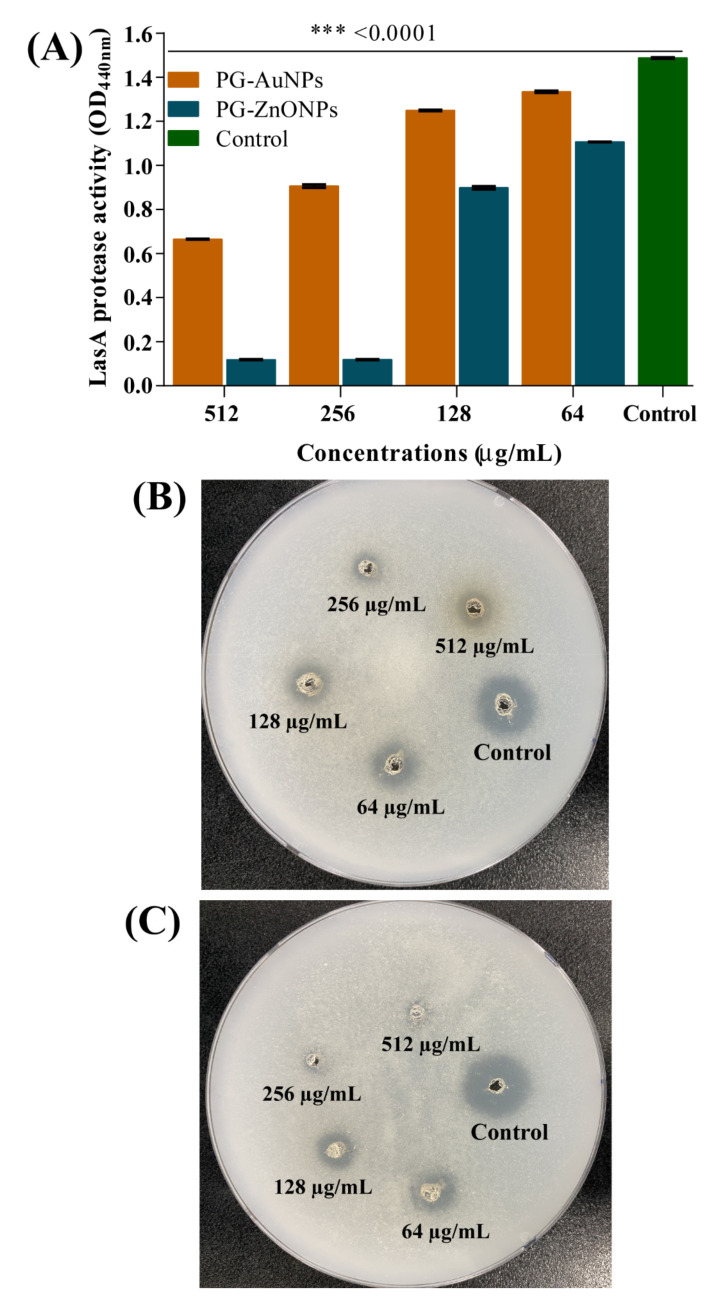
Protease inhibitory effect of PG-AuNPs and PG-ZnONPs towards *P*. *aeruginosa*. (**A**) Inhibition of LasA protease activity by PG-AuNPs and PG-ZnONPs, (**B**) protease inhibition by PG-AuNPs on the casein agar plate, and (**C**) protease inhibition by PG-ZnONPs on the casein agar plate. *** denotes significance at *p* < 0.0001.

The effect of PG-AuNPs and PG-ZnONPs on protease production was examined by spectrophotometer as well as on the agar plate. In the case of spectrophotometric-based analysis, the digestion of azocasein protein was examined colorimetrically. The result was concentration-dependent inhibition of LasA protease activity by PG-AuNPs and PG-ZnONPs (Figure 11A). The maximum LasA protease inhibition by PG-AuNPs (55.3%) and PG-ZnONPs (92%) was found to be at 512 and 256 μg/mL. Similarly, as a qualitative assay, protease activity was also inhibited by PG-AuNPs and PG-ZnONPs, as examined by the digestion of casein on an agar plate (Figure 11B,C).

**Figure 12:**
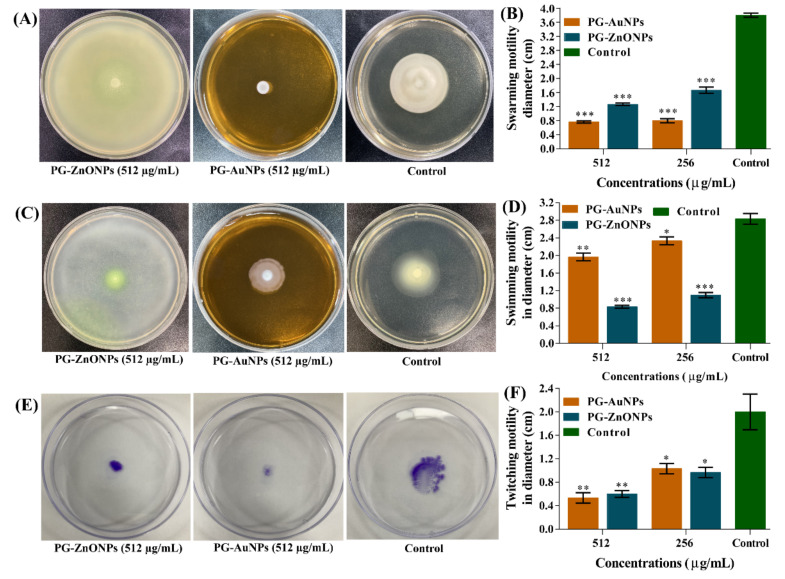
Motility inhibition properties of PG-AuNPs and PG-ZnONPs towards *P*. *aeruginosa*. (**A**) Representative agar plates showing the swarming motility, (**B**) graph showing the values of swarming motility in diameter, (**C**) representative agar plates showing the swimming motility, (**D**) graph showing the values of swimming motility in terms of diameter, (**E**) representative agar plates showing the twitching motility, and (**F**) graph showing the values of twitching motility in terms of diameter. ***, **, and * denote significance at *p* < 0.0001, *p* < 0.01, and *p* < 0.05.

Different types of motility such as swarming, swimming, and twitching in *P*. *aeruginosa* play important roles in sensing biotic/abiotic surfaces, surface attachment, and biofilm formation [10]. The swarming motility of *P*. *aeruginosa* was significantly inhibited by PG-AuNPs and PG-ZnONPs. The swarming motility at a concentration of 512 μg/mL of PG-AuNPs and PG-ZnONPs was found to be 79.8% and 66.6%, respectively (Figure 12B). Similarly, the swimming motility was inhibited significantly by PG-AuNPs and PG-ZnONPs, and the inhibition was at its maximum at a concentration of 512 μg/mL (Figure 12D). The pili-mediated motility, i.e., twitching, was also significantly inhibited, and a maximum inhibition of 73.3% (by PG-ANPs) and 70% (by PG-ZnONPs) was observed at a concentration of 512 μg/mL (Figure 12F). The representative agar plates showing different types of motility are displayed in Figure 12A,C,E.

### 2.5. In Vitro Cytotoxicity Assay

**Figure 13:**
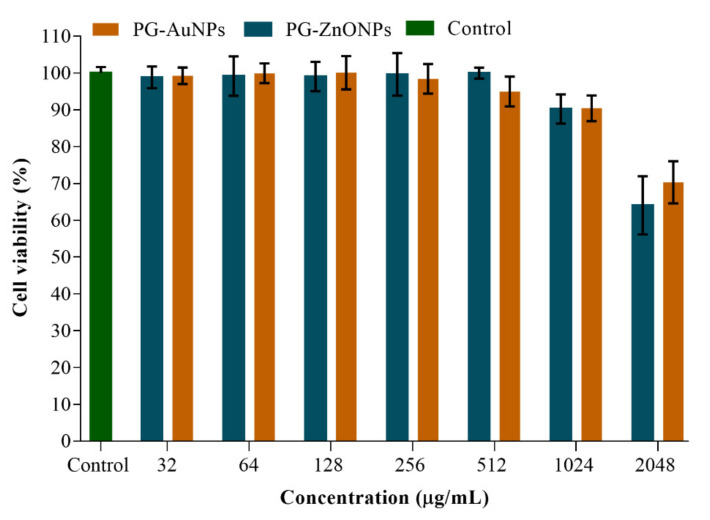
Cell cytotoxicity assay of PG-AuNPs and PG-ZnONPs towards a RAW 264.7 animal cell culture.

The in vitro cytotoxicity of PG-AuNPs and PG-ZnONPs against the macrophage cell line RAW 264.7 has been studied (Figure 13). These NPs did not exhibit cytotoxicity at concentrations ranging from 32 to 1024 μg/mL, but at 2048 μg/mL cytotoxicity was detected (Figure 13). In the current work, the antibiofilm and antivirulence characteristics of PG-AuNPs and PG-ZnONPs were reported up to a sub-MIC value of 512 μg/mL, which is two-fold lower than the cytotoxicity concentration (2048 μg/mL).

## 3. Discussion

With the advancement of nanotechnology and the extensive breadth of its applications in biology and medicine, several researchers across the globe have been actively working to synthesize nanomaterials to treat infectious diseases [36]. It has been discovered that nanoparticles offer numerous advantages due to their small size scale, comparable to ligands, receptors, effectors, channels, and nucleic acids, and may even be engineered to achieve a specific physiological impact [37]. However, their preclinical use is challenging due to the range of materials used, unique surface properties, reactivity, and numerous other multifunctional characteristics in vivo [37,38]. As a result, it is always encouraging to use biological materials to synthesize new and novel nanoparticles. Several types of nanoparticles have been synthesized in current and previous research by utilizing natural products derived from plants, bacteria, algae, and fungi [14,39]. However, the majority of the time, the natural product employed for nanoparticle synthesis is a cell extract [39]. Naturally derived extracts contain a variety of biologically active compounds, such as phenolic compounds (plants contain over 8000 phenolic compounds) [40], making it difficult to pinpoint the exact active component involved in nanoparticle synthesis. The current study used PG, a naturally derived phenolic compound, to synthesize gold and zinc oxide nanoparticles. Several instrumental techniques were used to characterize the synthesized PG-AuNPs and PG-ZnONPs fully. The formation of a wine-red color and the appearance of a white precipitate in the reaction mixtures indicate that PG-AuNPs and PG-ZnONPs have been completely synthesized, as previously described [13,41].

Furthermore, the synthesized NPs have distinct absorption spectra, such as 480 nm (PG-AuNPs) as well as 315 nm and 359 nm (PG-ZnONPs), but these are not identical to the previously described AuNPs and ZnONPs [13,41]. The slight variation in absorption might be attributed to differences in the reaction mixture conditions and composition. As previously reported, similar characteristic vibration bands were found in the FTIR spectra of PG [42]. Interestingly, some of the vibration bands have also been seen in the FTIR spectra of PG-AuNPs and PG-ZnONPs, indicating an interaction between PG and nanoparticles, which is consistent with previously reported NPs that were synthesized using different natural materials [41]. Although the average size of PG-AuNPs is not comparable to earlier studies, because varying sizes have been reported, the shape of PG-AuNPs is spherical, as is the case for several other AuNPs [41,43]. The shape of PG-ZnONPs was hexagonal, with an average size of 52.7 ± 3.8 nm, which is similar to the previously synthesized ZnONPs using *Aloe vera* peel extract [44]. The zeta potential measurement of PG-AuNPs and PG-ZnONPs revealed average surface charge values of −33.73 ± 0.25 mV and −35.62 ± 0.30 mV, indicating that these NPs are physically stable, as has been previously reported [45,46]. The appearance of distinct diffraction peaks of PG, PG-AuNPs, and PG-ZnONPs, as previously reported for AuNPs and ZnONPs, indicating the successful synthesis of these nanoparticles [13,41,47]. Thus, based on various instrumental characterizations, it is demonstrated that PG-AuNPs and PG-ZnONPs were successfully synthesized by employing PG, a natural phenolic compound.

*P*. *aeruginosa* is a common cause of nosocomial infections in immunocompromised, cystic fibrosis, severe burns, and infected respiratory patients [2,5]. As reviewed earlier by Pang et al. [5], *P*. *aeruginosa* has a number of intrinsic and acquired antibiotic resistance mechanisms. However, adaptive resistance mechanisms, such as biofilm formation and the generation of antibiotic-tolerant persister cells, as well as the production of several virulence factors have been the major cause of pathogenesis and chronic infection [9,48]. The antibiofilm properties of PG-AuNPs and PG-ZnONPs against *P*. *aeruginosa* were found to be concentration-dependent, which is consistent with previously reported inhibitory activity by NPs [43,49]. The antibiofilm efficacy was further verified by SEM imaging of biofilm architecture, where the PG-AuNP- and PG-ZnONP-treated samples demonstrated complete inhibition of attachment to the surface of the nylon membrane when compared to the control, which is consistent with previous studies [43]. Similarly, a significant reduction in fluorescence intensity in PG-AuNP- and PG-ZnONP-treated cells compared to the control is consistent with previous observations [43,50]. Since they contain antibiotic-tolerant persister cells, eradicating established mature *P*. *aeruginosa* biofilms is another potential strategy for controlling recurrent infection [11]. In the maturation stage of biofilm, cells are dispersed and become free-floating planktonic cells that can reinfect the host cells [51]. The chronic wound has been identified as a microenvironment that promotes mature biofilm formation and results in antimicrobial treatment failure [52]. Furthermore, due to the presence of a thick EPS matrix in the mature biofilm, a high concentration of the drug is required to eradicate it [53]. Sub-MIC and above-sub-MIC concentrations of PG-AuNPs and PG-ZnONPs significantly eradicate mature biofilm. Furthermore, the eradication of mature biofilm by these NPs occurred in a concentration-dependent manner, which is consistent with previous observations [41,54].

To date, there is no conclusive mechanism of nanoparticle biofilm inhibitory properties; however, some reports show that there is inhibition in gene expression associated with biofilm formation, altering cell membrane permeability and the production of reactive oxygen species that can disrupt cellular function [49,54,55,56]. In addition to the formation of biofilm as an adaptive resistance mechanism, *P*. *aeruginosa* pathogenicity and chronic infection have been reported as a result of the production of an array of virulence factors involved in a variety of functions, such as motility (flagella-mediated swarming and swimming as well as type IV-pili-mediated twitching motility) and host cell damage (e.g., siderophore, protease, hemolysin, pyocyanin, exotoxin/endotoxin A, and rhamnolipid) [57]. As previously documented, quorum sensing (QS) regulates the synthesis of some virulence factors, such as hemolytic activity, pyocyanin, pyoverdine, and protease activity, of *P*. *aeruginosa* [58]. As a result, attenuating the pathogen’s virulence characteristics has been identified as another potential strategy that involves the pathogen’s disarmament by attenuating the production of virulence factors [59,60]. The production of virulence factors, such as pyoverdine, pyocyanin, hemolysin, protease, and rhamnolipid, of *P*. *aeruginosa* has been significantly reduced by PG-AuNPs and PG-ZnONPs, which is consistent with earlier studies [43,49]. Except for pyocyanin and rhamnolipid production, the rest of the virulence factor production by PG-AuNPs and PG-ZnONPs was shown to be inhibited in a concentration-dependent manner. Similarly, earlier studies found significant inhibition of pyocyanin and rhamnolipid production rather than concentration-dependent inhibition [43,50,61]. Future work will be required to investigate why these NPs did not inhibit *P*. *aeruginosa* pyocyanin and rhamnolipid production in a concentration-dependent manner.

These PG-AuNPs and PG-ZnONPs strongly reduced all forms of motility, including swarming, swimming, and twitching, suggesting that they may have virulence-attenuating characteristics (Figure 12). Although similar antivirulence effects of AuNPs and ZnONPs that were synthesized using natural extracts have been reported [62,63], information on NPs synthesized from pure natural compounds is limited. As a result, this study encourages researchers to investigate the synthesis of all forms of metallic and metal oxide nanoparticles using a purely natural compound for various biological functions. Several nanoparticles have broad antibacterial and antibiofilm properties against Gram-positive and Gram-negative bacteria, as well as fungal pathogens [25,64,65]. It has also been discovered that *P*. *aeruginosa* is responsible for mixed microbial infections due to the co-existence of multiple bacterial (Gram-positive and Gram-negative) and fungal (e.g., *Candida albicans* and *Aspergillus fumigatus*) pathogens in addition to the formation of polymicrobial biofilms [66]. Hence, future studies are required to investigate the antibiofilm properties of PG-AuNPs and PG-ZnONPs towards polymicrobial biofilms.

## 4. Materials and Methods

### 4.1. Bacterial Strains, Culture Media, and Chemicals

*Pseudomonas aeruginosa* PAO1 (KCTC 1637) was purchased from the Korean Collection for Type Cultures (KCTC, Daejeon, Korea). Tryptic soy broth (TSB; Difco Laboratory Inc., Detroit, MI, USA) was utilized as the culture medium for the growth of *P*. *aeruginosa*. Gold (III) chloride trihydrate (CAS # 16961-25-4; purity 99%), phloroglucinol (CAS # 108-73-6; purity ≥ 99%), zinc acetate (Zn(CH_3_-COO)_2_.2H_2_O) (CAS # 5970-45-6), and commercial ZnONPs (CAS # 1314-13-2; purity > 97% and particle size < 50 nm) were purchased from Sigma-Aldrich Co. (St. Louis, MO, USA). The composition of minimal salt medium (MSM), per liter, includes Na_2_HPO_4_ (4.0 g), KH_2_PO_4_ (2.0 g), (NH_4_)_2_SO_4_ (0.8 g), MgSO_4_ (0.8 g), and 1 mL trace element solution (TES) [67]. The composition of the TES, per liter, includes (NH_4_)_6_Mo7O_24_.4H_2_O (0.05 g), Al(OH)_3_ (0.1 g), BaCl_2_ (0.05 g), CoCl_2_.6H_2_O (0.1 g), H_3_BO_3_ (0.5 g), KI (0.05 g), LiCl (0.05 g), MnSO_4_.4H_2_O (0.08 g), NiSO_4_.6H_2_O (0.1 g), SnCl_2_ (0.05 g), and ZnSO_4_.7H_2_O (0.1 g). The pH of MSM was 7.2.

### 4.2. Synthesis of Phloroglucinol Gold and Zinc Oxide Nanoparticles

The synthesis of phloroglucinol-gold nanoparticles (PG-AuNPs) and phloroglucinol-zinc oxide nanoparticles (PG-ZnONPs) was carried out with some modifications to the procedure previously reported [13,41,68]. The synthesis of PG-AuNPs began with the dissolution of PG (0.5%) in sterile, deionized water at a temperature of 50–60 °C under stirring conditions. Using 0.1 M NaOH, the pH of the PG solution was adjusted to 8.5. After one hour of stirring, an aqueous solution (200 mL) of 1 mM of gold (III) chloride trihydrate (HAuCl_4_.3H_2_O) was added to the PG solution. The final pH of the solution was adjusted to 8.5–9.0, and the mixture was continuously stirred for 2 h at 60 °C. When the color of the solution changed from yellow to dark wine-red, the presence of synthesized PG-AuNPs was verified. The PG-AuNP solution was frozen at −70 °C and then freeze-dried with a freeze-dryer (FD8518, ilShinBiobase Co. Ltd., Yangju-si, Korea). The PG-ZnONPs were prepared by dissolving 20 mM of Zn(CH_3_-COO)_2_.2H_2_O in deionized water and stirring it for 1 h at 70 °C. The PG (0.5%; prepared in deionized water) solution was progressively added to the Zn(CH_3_-COO)_2_.2H_2_O solution. After 1 h of continuous stirring at 70 °C, 0.1 N NaOH was added dropwise to this mixture till the fading white solution appeared. The solution was centrifuged at 13,000 rpm for 25 min, and the white precipitate was recovered after three washes with deionized water. A freeze-dryer was also used to freeze-dry the PG-AuNP pellets. The steps involved in the synthesis of PG-AuNPs and PG-ZnONPs are shown schematically in Figure 1.

### 4.3. Instrumental Characterization of PG-AuNPs and PG-ZnONPs

Apart from the appearance of a dark wine-red color (PG-AuNPs) and a white precipitate (PG-ZnONPs) as preliminary indications, the formation of these NPs was also validated by analyzing characteristic UV–Vis absorption spectra using a microplate reader (BioTek, Winooski, VT, USA) in the region of 200 to 800 nm. Several other instrumental analyses for the characterization of these NPs were carried out, as has been reported previously [41]. Fourier-transform infrared spectrometer (FTIR, JASCO (FT-4100), Tokyo, Japan) was used to characterize the PG-AuNPs and PG-ZnONPs at a frequency ranging from 4000 to 400 cm^−1^. The shape of PG-AuNPs and PG-ZnONPs was determined using field emission transmission electron microscopy (FETEM; JEM-F200, JEOL, Tokyo, Japan). The size and distribution of each NP were determined by dynamic light scattering (DLS) using a particle analyzer Litesizer 500 (Anton Paar, GmbH). Similarly, the particle analyzer was also used to determine the zeta potential of PG-AuNPs and PG-ZnONPs. The elemental composition of PG-AuNPs and PG-ZnONPs was determined by an energy-dispersive X-ray spectrometer (EDS; TESCAN, Brno, Czech Republic), VEGA II LSU). An X-ray diffractometer (XRD; X-ray diffractometer, Rigaku, Tokyo, Japan, Ultima IV) was used to determine the crystalline nature of PG-AuNPs and PG-ZnONPs. The characterized PG-AuNPs and PG-ZnONPs were used to check their antibiofilm and antivirulence activities towards *P*. *aeruginosa*.

### 4.4. Minimum Inhibitory Concentration (MIC) of PG-AuNPs and PG-ZnONPs

The minimal inhibitory concentration of PG, PG-AuNPs, PG-ZnONPs, and commercial ZnONPs (C-ZnONPs) against *P*. *aeruginosa* was determined using the microbroth dilution technique, as has been described previously [41]. After 12 h of incubation, the *P*. *aeruginosa* growing culture was diluted (1:100) in TSB and put in a 96-well polystyrene microplate (SPL Life Sciences Co., Ltd., Pocheon-si, Korea). These cultures were also treated individually with different concentrations of PG-AuNPs and PG-ZnONPs (ranging from 64 to 2048 μg/mL). Using a microplate reader (BioTek, Winooski, VT, USA), the microplate was incubated at 37 °C for 24 h under shaking conditions (567 cycles per minute; cpm). The optical density (OD_600_) of the growing cell culture was measured after 24 h of incubation. The OD_600_ values of NP-treated cell cultures were subtracted from the OD_600_ value of the control (TSB containing only different concentrations of NPs). The experiment was conducted three times in triplicate.

### 4.5. Bacterial Biofilm Inhibition and Eradication Assays

The inhibitory action of PG-AuNPs and PG-ZnONPs on bacterial biofilms was investigated in the same manner as previously described [41]. A 96-well microplate was used to investigate the formation of biofilm and the inhibitory impact of NPs. The diluted (1:100, equivalent to an OD_600_ value of 0.05) cell culture (300 µL) obtained from overnight growth at 37 °C was put on a 96-well microplate with various concentrations of NPs ranging from 64 to 512 μg/mL. The microplate was incubated at 37 °C for 24 h without shaking. A microplate reader was used to determine the OD_600_ values of the total cells (planktonic as well as adhered). The free-floating cells were removed from the microplate wells, and the biofilm cells adhering to the surface were washed three times with distilled water. For 20 min the cells were stained with aqueous crystal violet (0.1%). The stained biofilm cells were rinsed three times with distilled water and air-dried after removing the leftover crystal violet from each well. The stained cells from each well were dissolved in 95% ethanol before being quantified at 570 nm.

To eradicate mature biofilm using NPs, we first allowed a mature biofilm to be established in a 96-well microplate. The diluted cell culture (300 µL) was put in a microplate and incubated at 37 °C for 24 h. After incubation, the free-floating planktonic cells in the microplate were discarded, and the adherent biofilm cells were washed three times with sterile TSB. Different concentrations of NPs (varying from 64 to 1024 μg/mL) prepared in sterile TSB were put in the well containing mature biofilms. The microplate was incubated at 37 °C for 24 h. The growth medium from each well that contained free-floating planktonic cells was discarded, and adherent biofilm cells were washed thrice with distilled water. The biofilm cells were stained using crystal violet (0.1%), and OD_570_ values were used to estimate the NP eradication effectiveness. Similarly, the eradication efficiency of PG-AuNPs and PG-ZnONPs against *P*. *aeruginosa* mature biofilm was determined by measuring colony-forming units (CFUs), as has been previously described [50]. In brief, the free-floating planktonic cells from biofilm matured for 24 h were removed, and the adherent cells were washed three times with a sterile TSB medium. Different concentrations of NPs (prepared in sterile TSB), ranging from 64 to 1024 μg/mL, were added to the microplate containing adherent cells and incubated at 37 °C for 24 h. After discarding the planktonic cells, the adhering cells were washed three times with sterile TSB, scraped with sterile pipette tips, and resuspended in 300 μL of TSB media. These cell suspensions were serially diluted in fresh TSB up to a dilution of 10^−8^. The diluted cell culture (100 μL) was spread-plated onto a TSA plate and incubated at 37 °C overnight. The colony on the agar plate was counted, and the CFUs were calculated. The experiment was conducted three times in triplicate.

### 4.6. Examination of Biofilm Architecture Using a Scanning Electron Microscope and Fluorescence Microscope

Scanning electron microscopy (SEM) was carried out to examine the biofilm architecture affected by the exposure to NPs, as described earlier [50]. The diluted cell culture (300 µL) was allowed to form biofilm on the surface of the nylon membrane (0.5 × 0.5 cm) placed in the 24-well microplate. These cell cultures were also treated with PG-AuNPs and PG-ZnONPs separately, and incubated at 37 °C for 24 h. After incubation, the biofilm cells on the nylon surface were directly fixed using glutaraldehyde (2.5%) and formaldehyde (2%) overnight at 4 °C. Unattached (planktonic) cells were removed from the well, and the nylon-surface-attached biofilm cells were gently washed thrice with phosphate-buffered saline (pH of 7.4). The biofilm cells were dehydrated using an increasing concentration of ethanol. Finally, the biofilm cells on the nylon membrane surface were freeze-dried using a freeze-dryer. The scanning of the biofilm architecture was done with a TESCAN scanning electrom microscope (Vega II LSU, Czech Republic). Furthermore, the biofilm inhibitory effect of both PG-AuNPs and PG-ZnONPs was evaluated by viewing the cells under a fluorescent microscope (Leica DMI300B Microsystems, Wetzlar, Germany) [50]. In brief, a diluted (1:100) cell culture of *P*. *aeruginosa* cells grown overnight was allowed to form biofilm on a glass coverslip surface that was placed on a 6-well microplate in the presence and absence of NPs. After 24 h of incubation at 37 °C, the biofilm cells on the glass surface were washed three times with PBS (pH 7.4), followed by 10 min of staining with acridine orange (10 μg/mL). The cells were visualized using a microscope after being washed with PBS.

### 4.7. Assays for the Virulence Properties

The hemolytic activity was determined using sheep red blood cells (RBCs; MBcell Ltd., Seoul, Korea) in accordance with previous reports [50]. In brief, diluted RBCS (950 µL) was seeded with NP-treated and non-treated *P*. *aeruginosa* cell cultures (50 µL) and incubated for 1 h at 37 °C under shaking conditions (250 rpm). The OD_543_ of the supernatant was measured to assess RBC hemolysis. The colorimetric approach was used to examine the effect of NPs on pyocyanin production [69]. The NP-treated and non-treated cell cultures were centrifuged (13,000 rpm for 25 min) and the cell-free supernatant was recovered. The extraction was carried out by adding 3 mL of chloroform to the supernatant (5 mL) and vortexing it for 5 min. The chloroform layer was collected in a separate tube and mixed with 0.2 N HCl. The pink-colored solution that appeared in the top layer was collected and quantified by measuring the OD at 520 nm. The impact of PG-AuNPs and PG-ZnONP on pyoverdine production was investigated by cultivating the bacterial cell in the iron-free MSM containing 2% sodium succinate and different NP concentrations. The OD at 405 nm was used to quantify the pyoverdine in the cell-free supernatant. The effect of NPs on protease activity was studied using a spectrophotometer in a liquid medium and on a Bacto agar plate containing casein, as previously described but with a slight modification [58]. After overnight incubation with varying doses of NPs, cell cultures were centrifuged and filter-sterilized to recover the released LasA protease. The protease activity was determined by combining supernatant (150 µL) with 250 µL of azocasein (2%, prepared in 50 mM Tris-HCl, pH 7.8) in a tube and incubating it at 37 °C for 4 h. The protease reaction was stopped for 15 min using 10% trichloroacetic acid. Furthermore, the whole process was neutralized with 0.1 N NaOH. The supernatant from the reaction mixture was recovered by centrifuging it (13,000 rpm for 10 min), and the OD at 440 nm was measured. The protease inhibition activity of NPs on the agar plate was examined using a Bacto agar plate containing 5% casein powder. The casein–agar plate hole was filled with the collected cell-free supernatant from the culture that had been treated with varying concentrations of NPs. These agar plates were incubated at 37 °C for 24 h. The cleared zone around the hole was considered positive for protease activity, and a comparison was made between the treated and untreated groups (control). The effect of NPs on various forms of motility, such as swarming, swimming, and twitching, was studied using the previously described procedure [58]. Bacto agar (0.5%), glucose (0.5%), and casamino acid (0.4%) were used to produce the medium for swarming motility in Luria–Bertani (LB) broth. Bacto agar (0.3%), tryptone (1%), and NaCl (0.25%) were used to make the medium for the swimming assays.

The medium composition for twitching motility was glucose (30 mM), casamino acid (0.2%), and Bacto agar (1.5%), which was also produced in LB broth. The cell culture grown overnight (5 µL) was put on the surface of swarming and swimming agar plates. In the case of twitching motility assays, the cell culture (5 µL) was placed on the surface of a Petri dish with the help of a sterile toothpick before pouring the agar media. The effect of NPs on swarming and swimming motility was investigated by measuring the cell diameters (cm) traversed. The cell motility was evaluated in the instance of twitching by staining with 0.1% crystal violet. The experiment was conducted three times in triplicate.

### 4.8. In Vitro Cytotoxicity Assay

MTT (3,4,5-dimethylthiazol-2-yl)-2-5-diphenyl tetrazolium bromide dye was used to test the in vitro cytotoxicity of PG-AuNPs and PG-ZnONPs against the mouse macrophage cell line RAW 264.7 [70]. The experiment was carried out by adding different concentrations of NPs (ranging from 32 to 2048 μg/mL) to a 96-well microtiter plate containing pre-incubated RAW 264.7 cells for 24 h. The titer plate was incubated at 37 °C for 24 h under static conditions. The cells were rinsed with PBS (pH of 7.4), and a fresh culture medium containing MTT dye (1 μg/mL) was added. The microplate was then incubated at 37 °C for 3 h with CO_2_ supplied (5%). Following incubation, the media was discarded and DMSO was added, followed by another 30 min of incubation at 37 °C. The fluorescence was quantified by measuring the OD at 570 nm using a microplate reader (Gen 5^TM^ ELISA Bio Tek, Winooski, VT, USA). The experiment was conducted three times in triplicate.

### 4.9. Statistical Analysis

All graphs were created using GraphPad Prism 7.0 (GraphPad Software Inc., San Diego, CA, USA). Furthermore, each experimental dataset was statistically analyzed using one-way ANOVA followed by Dunnett’s multiple comparisons test. *** *p* < 0.0001, ** *p* < 0.01, and * *p* < 0.05 were considered significant. Each experiment was repeated at least three times, and the present results were the mean ± standard error of the three replicates.

## 5. Conclusions

This study demonstrated the green synthesis of AuNPs and ZnONPs utilizing PG, a natural phenolic compound. Several instruments were used to characterize the synthesized NPs, which were PG-AuNPs and PG-ZnONPs. The biological activity of PG-AuNPs and PG-ZnONPs against *P*. *aeruginosa*, an opportunistic pathogen that causes chronic and acute infection in humans, was also investigated. The PG-AuNPs and PG-ZnONPs inhibited biofilm formation and eradicated mature *P*. *aeruginosa* biofilms in a concentration-dependent manner. PG-AuNPs and PG-ZnONPs significantly reduced the production of several virulence factors in *P*. *aeruginosa*, including hemolytic activity, protease activity, rhamnolipid production, pyocyanin production, and pyoverdine production. Furthermore, the PG-AuNPs and PG-ZnONPs efficiently reduced all forms of motility in *P*. *aeruginosa*, including swarming, swimming, and twitching. The antibiofilm and antivirulence characteristics of PG-AuNPs and PG-ZnONPs were reported at concentration, which is two-fold lower than the cytotoxicity concentration. As a result, these NPs can be employed as biocompatible drugs to treat *P*. *aeruginosa* infection. Thus, based on the findings of the present study, it can be concluded that metallic and metal oxide nanoparticles may be synthesized from a single, pure phenolic compound as a potential antibiofilm and antivirulence agent against bacterial pathogens. Future research will be needed to elucidate the molecular mechanisms by which PG-AuNPs and PG-ZnONPs inhibit biofilm formation and virulence. The present study provides a novel perspective into the use of pure, naturally isolated compounds rather than extracts for the synthesis of potential nanoparticle drugs. Since *P*. *aeruginosa* and other bacterial pathogens (e.g., *Staphylococcus aureus* and *Acinetobacter baumannii*) have been reported to form biofilm in the sites of burn wounds, which has become one of the major causes of failure in the treatment of wound infection [52], future study is therefore required to investigate the biofilm eradication efficiency of PG-AuNPs and PG-ZnONPs using in vivo model organisms.
